# Skeletal muscle mass at C3 may not be a strong predictor for skeletal muscle mass at L3 in sarcopenic patients with head and neck cancer

**DOI:** 10.1371/journal.pone.0254844

**Published:** 2021-07-19

**Authors:** Joon-Kee Yoon, Jeon Yeob Jang, Young-Sil An, Su Jin Lee

**Affiliations:** 1 Department of Nuclear Medicine and Molecular Imaging, Ajou University School of Medicine, Suwon, Republic of Korea; 2 Department of Otolaryngology, Ajou University School of Medicine, Suwon, Republic of Korea; IRCCS Ospedale Policlinico San Martino, Genova, ITALY

## Abstract

**Purpose:**

To evaluate the feasibility of using skeletal muscle mass (SMM) at C3 (C3 SMM) as a diagnostic marker for sarcopenia in head and neck cancer (HNC) patients.

**Methods:**

We evaluated 165 HNC patients and 42 healthy adults who underwent ^18^F-fluorodeoxyglucose positron emission tomography/computed tomography scans. The paravertebral muscle area at C3 and skeletal muscle area at L3 were measured by CT. Pearson’s correlation was used to assess the relationship between L3 and C3 SMMs. The prediction model for L3 SMM was developed by multiple linear regression. Then the correlation and the agreement between actual and predicted L3 SMMs were assessed. To evaluate the diagnostic value of C3 SMM for sarcopenia, the receiver operating characteristics (ROC) curves were analyzed.

**Results:**

Of the 165 HNC patients, 61 (37.0%) were sarcopenic and 104 (63.0%) were non-sarcopenic. A very strong correlation was found between L3 SMM and C3 SMM in both healthy adults (r = 0.864) and non-sarcopenic patients (r = 0.876), while a fair association was found in sarcopenic patients (r = 0.381). Prediction model showed a very strong correlation between actual SMM and predicted L3 SMM in both non-sarcopenic patients and healthy adults (r > 0.9), whereas the relationship was moderate in sarcopenic patients (r = 0.7633). The agreement between two measurements was good for healthy subjects and non-sarcopenic patients, while it was poor for sarcopenic patients. On ROC analysis, predicted L3 SMM showed poor diagnostic accuracy for sarcopenia.

**Conclusions:**

A correlation between L3 and C3 SMMs was weak in sarcopenic patients. A prediction model also showed a poor diagnostic accuracy. Therefore, C3 SMM may not be a strong predictor for L3 SMM in sarcopenic patients with HNC.

## Introduction

Sarcopenia is characterized by low skeletal muscle mass (SMM) combined with low muscle strength or low physical performance, and is prevalent in cancer patients. Sarcopenia has been suggested as a negative prognostic factor in patients with malignancies, including head and neck cancer (HNC) [1–5]. Therefore, evaluation of the presence of sarcopenia in oncological patients is important in clinical practice.

Several methods are available for assessing SMM, including dual-energy X-ray absorptiometry, bioelectric impedance analysis, magnetic resonance imaging (MRI) and computed tomography (CT). In particular, MRI and CT are considered precise imaging modalities that can distinguish fat and muscle from other soft tissues [6–10]. Skeletal muscle area (SMA) at the third lumbar vertebra (L3) is closely correlated with whole-body SMM [8]. Measurement of skeletal muscle index (SMI; height^2^-adjusted SMA) at the level of L3 is used for the diagnosis of sarcopenia [11]. CT performed during cancer staging procedures is an easy method for measuring SMA; intra- and inter-observer variability in skeletal muscle measurements is reportedly low when using CT [12].

However, CT imaging of L3 is not routinely performed in HNC patients. Several studies suggested that CT at the third cervical vertebra (C3) may be a cost-effective and reliable alternative to imaging at L3 because the SMM at L3 (L3 SMM) and C3 SMM show a strong correlation [13,14]. Several reports developed the prediction models using C3 SMM to estimate L3 SMM [3,13].

^18^F-fluorodeoxyglucose (FDG) positron emission tomography (PET)/CT is a valuable imaging modality for the management of HNC [15,16]. Torso PET/CT usually covers from head to thigh and can therefore be used to evaluate both C3 SMM and L3 SMM.

Although a strong correlation between L3 SMM and C3 SMM has been suggested in a study cohort involving HNC patients, their relationship in sarcopenic patients is unclear. Moreover, regional changes in muscle mass occur with aging [17]. We hypothesized that the change in L3 SMM may be more susceptible to aging or illness than C3 SMM, thus the relationship between L3 SMM and C3 SMM may not exist in sarcopenic patients. Therefore, we investigated the relationship between C3 SMM and L3 SMM in HNC patients and assessed the feasibility of using C3 SMM in sarcopenic patients.

## Materials and methods

### Patients

We retrospectively reviewed HNC patients between January 2010 and December 2013 in our hospital. The inclusion criteria were as follows: histologically proven HNC patients, patients who underwent pretreatment PET/CT then received appropriate treatment. The HNC patients without pretreatment PET/CT or refused treatment were excluded. Finally, a total of 165 HNC patients were included in this study. We additionally evaluated 42 healthy adults who underwent FDG PET/CT as part of a health screening program between September 2010 and September 2011 at our hospital. None of the healthy subjects had a history of malignancy. This retrospective study was approved by the Institutional Review Board of Ajou University hospital, and the requirement for informed consent was waived given the retrospective nature of the work (approval no. AJIRB-MED-MDB-20-209).

### FDG PET/CT acquisition

FDG PET/CT was performed using the Discovery STE PET/CT scanner (GE Healthcare, Milwaukee, WI, USA). All patients fasted for at least 6 h before FDG PET/CT; their blood glucose levels at the time of FDG injection were < 150 mg/dL. First, unenhanced CT images were acquired from the skull base to the upper thigh using the following parameters: 120 kV, 60 mA, 7.5 mm/rotation, 1 s/rev tube rotation time, 867 mm scan length and 60.9 s acquisition time. Immediately after CT, emission PET data were acquired from the thigh to the head for 3 min per frame in three-dimensional mode. Attenuation-corrected PET images (CT data were used for correction) were reconstructed using an ordered-subset expectation-maximization algorithm (20 subsets, 2 iterations).

### Measurement of skeletal muscle mass

**Fig 1:**
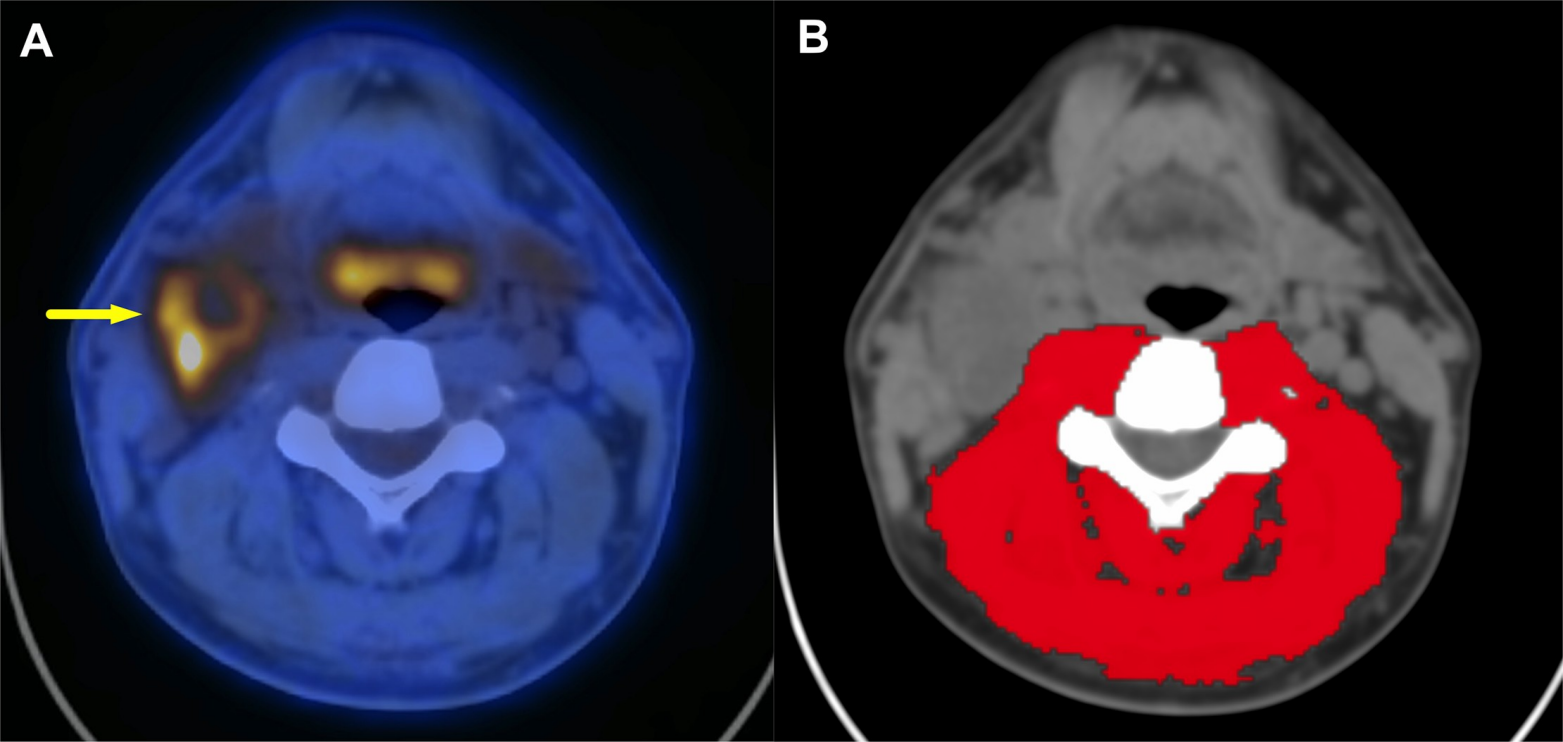
Representative fusion PET/CT (A) and CT (B) images used to measure the paravertebral muscle area at C3. A 57-year-old male patient with right tonsillar cancer. Metastatic lymphadenopathy is detected at right cervical levels II–III with invasion of the right sternocleidomastoid muscle.

The C3 SMM and L3 SMM were measured using OsiriX software (ver. 11.0.4; Pixmeo, Bernex, Switzerland) on each axial section of non-contrast-enhanced CT images obtained during PET/CT scanning. In accordance with previous reports, the Hounsfield unit threshold for identifying skeletal muscles was −29 to +150 [13,18,19]. When measuring the C3 SMM, we excluded the sternocleidomastoid (SCM) muscle because this muscle is frequently invaded by metastatic lymph nodes from HNC; therefore, we evaluated only the paravertebral muscle (PVM) area at C3 in this study (Fig 1). L3 SMM included the psoas muscles, erector spinae, quadratus lumborum, transversus abdominus, external and internal obliques and rectus abdominus.

The SMM measured at each vertebral level was normalized to height in meters squared and reported as the SMI (cm^2^/m^2^). According to an international consensus, sarcopenia is defined as an L3 muscle index < 55 cm^2^/m^2^ in men and < 39 cm^2^/m^2^ in women [11]. In this study, however, we defined sarcopenia as an L3 muscle index < 49 cm^2^/m^2^ for men and < 31 cm^2^/m^2^ for women based on a previous epidemiological study with a Korean population [20].

### Prediction model using C3 SMM

As most of the previous models adopted the sum of SCM muscle and PVM for C3 SMM in the prediction of L3 SMM [2,3,13,21], we developed our own prediction model using only PVM of HNC patients. Factors that are highly associated with sarcopenia were included as variables for the equation: age, sex, weight and PVM [22]. We coded males as 0 and as 1 for females. Multiple linear regression analysis was employed to compute the constant, the regression coefficients and t-statistics of independent variables and F-ratio of the analysis of variance table. T-values indicate the significance of each variable in this prediction model, while F-ratio tests whether the overall regression model is a good fit for the data. A *p*-value less than 0.05 was considered to be significant.


$$\begin{array}{l} Predicted\;L3=constant+b_{1}*C3\;PVM+b_{2}*BW+b_{3}*Age+b_{4}*Sex\\ \left(b_{1},\;b_{2},\;b_{3},\;b_{4}:\;regression\;coefficients\right) \end{array}$$

### Statistical analysis

Continuous variables are shown as means ± standard deviation (SD) and categorical variables as percentages. Differences between the two groups were compared using Student’s *t*-test for continuous variables and the chi-squared test for dichotomous variables. Pearson’s correlation was used to assess the relationship between C3 SMM and L3 SMM, and predicted L3 SMM and actual L3 SMM. Bland-Altman plots were drawn to assess the agreement between predicted L3 SMM and actual L3 SMM. The limit of agreement (LOA) and the mean of differences were calculated. The LOA was considered to show “good” agreement if the LOA was within 2 SD of actual L3 SMM, and it was “poor” if LOA was wider than 2SD of actual L3 SMM [23]. Analyses for the correlation and the agreement were also performed using SMIs, however, the standard for “good” or “poor” agreement was 3SD of actual L3 SMI. To evaluate the diagnostic value of C3 SMM for sarcopenia in patients with HNC, receiver operating characteristics (ROC) curve analysis with the measurement of area under the curve (AUC) was done using predicted L3 and L3 index. All analyses were performed using GraphPad Prism (ver. 8.4.3; GraphPad Software, San Diego, CA, USA) and MedCalc (ver. 19.5.3; MedCalc Software Ltd, Ostend, Belgium).

## Results

### Subjects characteristics

The clinical characteristics of the 165 HNC patients and 42 healthy subjects are summarized in Table 1. The HNC patients consisted of 142 male patients and 23 female patients aged 60.4 ± 12.2 years. The 42 healthy subjects consisted of 29 male adults and 13 female adults aged 52.5 ± 7.6 years. HNC patients had a significantly lower body weight and body mass index (BMI) compared with the healthy subjects. For HNC patients, tumors were staged as per tumor-node-metastasis (TNM) staging as suggested by the American Joint Committee on Cancer 7th edition. Forty (24.2%) patients had locally advanced cancer (T3-T4), 70 (44.2%) patients had lymph node metastasis (N1-N3), and 87 (52.7%) patients were advanced HNC (stage III-IV).

**Table 1:** Clinical characteristics of study subjects.

	HNC patients (n = 165)	Healthy adults (n = 42)	*p*-value
Age (year)	60.4 ± 12.2	52.5 ± 7.6	0.423
Male:female	142:23	29:13	0.020
Body weight (kg)	63.4 ± 11.6	69.4 ± 10.0	0.003
Body mass index (kg/m^2^)	23.2 ± 3.2	24.9 ± 2.1	0.001
T stage (T1/T2/T3/T4)	83/42/12/28	N.A	N.A
N stage (N0/N1/N2/N3)	92/18/53/2	N.A	N.A
Overall stage (I/II/III/IV)	59/19/26/61	N.A	N.A

N.A, not applicable.

Among the 165 HNC patients, 61 (37.0%) were sarcopenic at the time of cancer diagnosis, and 104 (63.0%) were non-sarcopenic based on the SMI at L3 (Table 2). All healthy subjects were non-sarcopenic. The sarcopenic patients with HNC were significantly older than the non-sarcopenic patients (63.9 ± 12.2 years vs. 58.3 ± 11.8 years, respectively, *p* = 0.004). All sarcopenic patients were male. The most frequent primary malignant lesions were in the oral cavity (30.8%) and larynx (30.8%) in non-sarcopenic patients and in the larynx (49.2%) and oropharynx (26.2%) in sarcopenic patients. Tumor, node, overall stage was not significant different between two groups.

**Table 2:** Comparison of clinical characteristics between patients with or without sarcopenia.

	Non-sarcopenia (n = 104)	Sarcopenia (n = 61)	*p-*value
Age (year)	58.3 ± 11.8	63.9 ± 12.2	0.004
Male:female	81:23	61:0	<0.0001
Primary lesion (%)			0.026
Oral cavity	32 (30.8)	11 (18.0)	
Nasopharynx	12 (11.5)	2 (3.3)	
Oropharynx	20 (19.2)	16 (26.2)	
Hypopharynx	8 (7.7)	2 (3.3)	
Larynx	32 (30.8)	30 (49.2)	
T stage (T1/T2/T3/T4)	56/28/7/13	27/14/5/15	0.225
N stage (N0/N1/N2/N3)	56/12/35/1	36/6/18/1	0.888
Overall stage (I/II/III/IV)	38/10/20/36	21/9/6/25	0.320
Body weight (kg)	65.5 ± 12.0	59.8 ± 9.8	0.002
Body mass index (kg/m^2^)	24.2 ± 3.0	21.6 ± 3.0	<0.0001

### Comparison of SMM among groups

**Fig 2:**
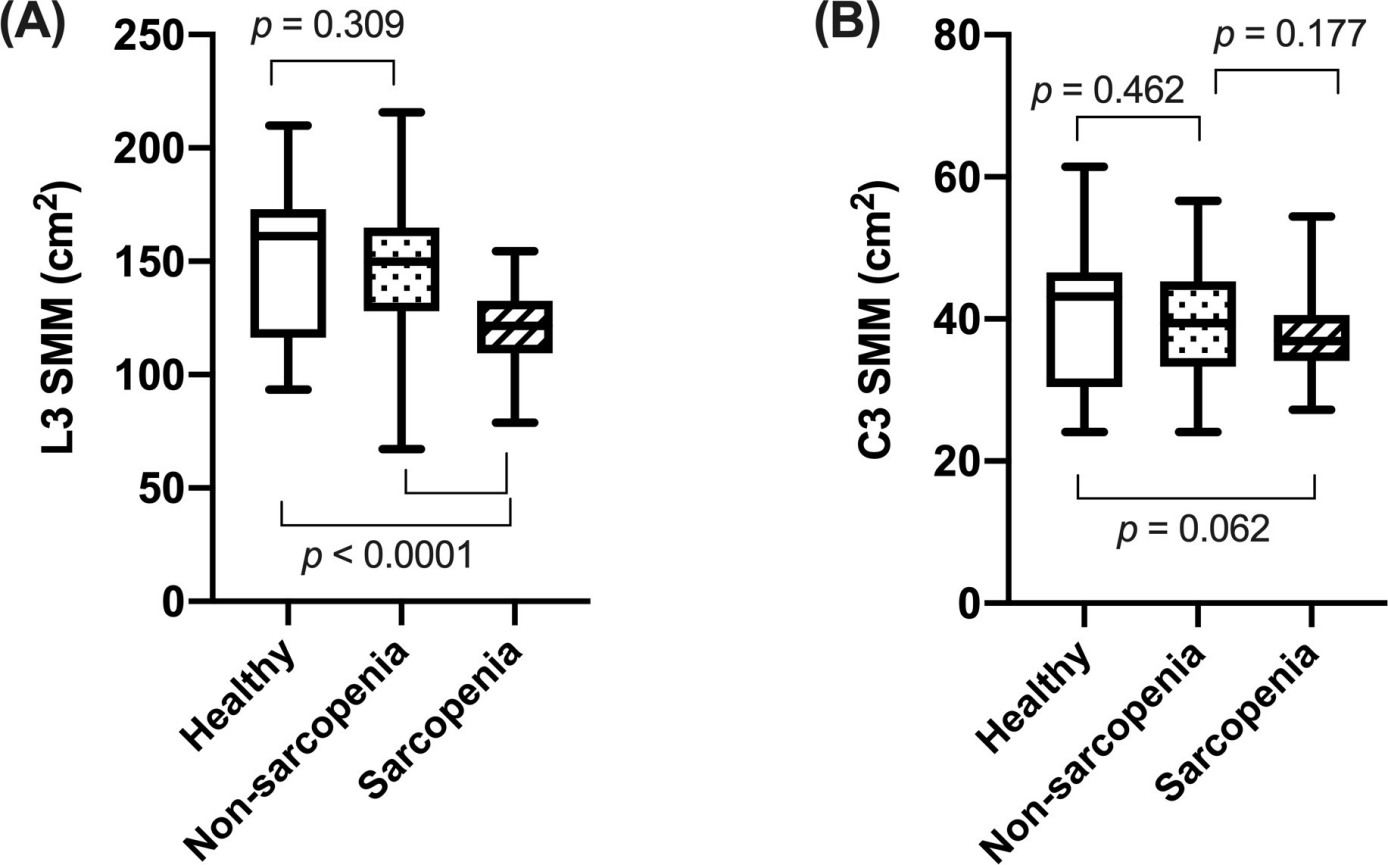
Comparison of skeletal muscle mass among the three groups. SMM, skeletal muscle mass.

Fig 2 shows the differences in SMM measured at each vertebral level among sarcopenic and non-sarcopenic HNC patients and healthy subjects.

The L3 SMM were not significantly different between healthy adults and non-sarcopenic patients (150.8 ± 33.4 cm^2^ and 144.8 ± 31.6 cm^2^, respectively, *p* = 0.309). As expected, these parameters were significantly different between healthy adults and sarcopenic patients (121.0 ± 17.2 cm^2^, *p* < 0.0001) and between non-sarcopenic patients and sarcopenic patients (*p* < 0.0001) (Fig 2A).

The mean C3 SMM were 40.4 ± 9.2 cm^2^ in healthy adults, 39.3 ± 8.2 cm^2^ in non-sarcopenic patients and 37.7 ± 5.5 cm^2^ in sarcopenic patients. The C3 SMM was not significantly different among the three groups (Fig 2B).

### Relationship between L3 SMM and C3 SMM

**Fig 3:**
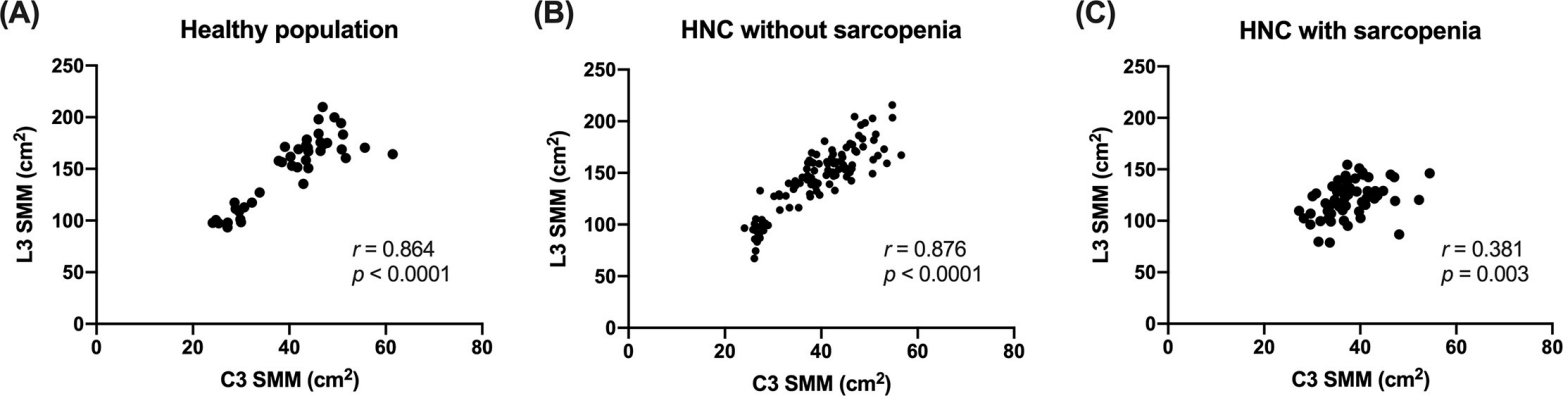
Association between the skeletal muscle mass at L3 and that at C3 in each group. SMM, skeletal muscle mass; HNC, head and neck cancer.

There was a very strong correlation between L3 SMM and C3 SMM in healthy subjects (r = 0.864, Fig 3A). A very strong correlation was also observed in the non-sarcopenic patients (r = 0.876, Fig 3B), whereas the correlation was fair in the sarcopenic patients (r = 0.381, Fig 3C).

**Fig 4:**
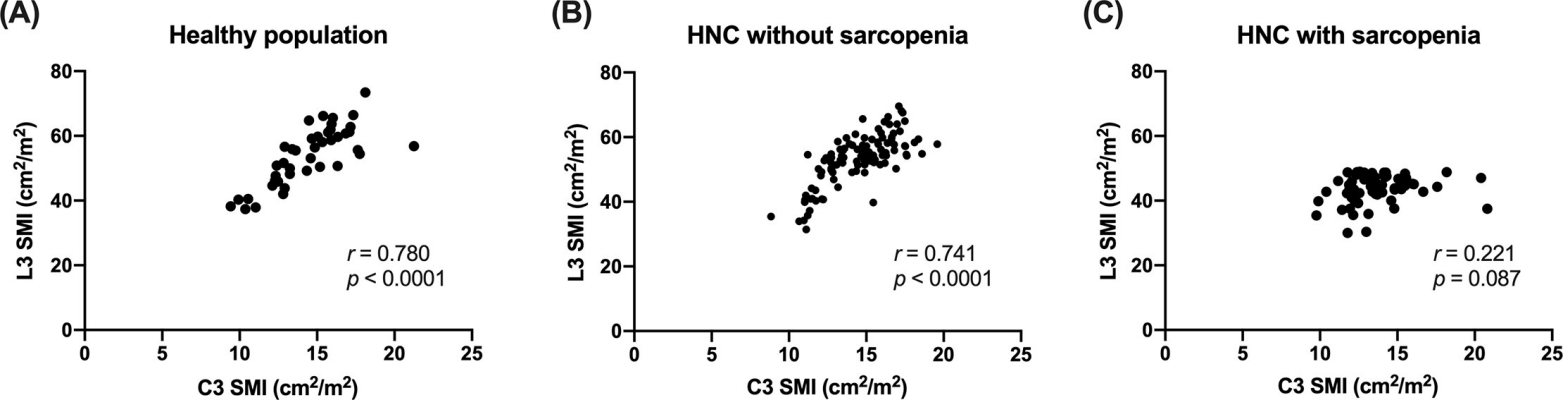
Association between the skeletal muscle index at L3 and that at C3 in each group. SMI, skeletal muscle index; HNC, head and neck cancer.

The subgroup analysis using L3 SMI and C3 SMI demonstrated similar results; a strong correlation was found in both healthy adults and non-sarcopenic patients (Fig 4A & 4B). However, the correlation was not significant in the sarcopenic patients (r = 0.221, Fig 4C).

### Prediction model and relationship between actual L3 SMM and predicted L3 SMM

Table 3 summarizes the statistical parameters by multiple regression analysis. All variables (age, sex, weight and PVM at C3) were significantly associated with L3 SMM (p < 0.05). We established a model with 4 variables as follows:

Predicted L3 = 45.9183 + 0.9736*C3 PVM+ 1.2863*BW– 0.4414*Age– 18.2159*Sex.

**Table 3:** Summary of regression analysis.

Independent variables	Coefficient	Standard error	t-value	*p*-value
(Constant)	45.9183			
Age	−0.4414	0.09806	−4.502	< 0.0001
BW	1.2862	0.1461	8.801	< 0.0001
PVM	0.9736	0.2631	3.701	0.0003
Sex	−18.2159	4.188	−4.35	< 0.0001

BW, Body weight; PVM, Paravertebral muscle.

Our prediction model showed a very strong positive correlation between actual SMM and predicted SMM at L3 in both non-sarcopenic HNC patients and healthy adults (r > 0.9), whereas the relationship was moderate in sarcopenic patients (r = 0.7633; Table 4) [24]. The results were similar for the prediction of SMI at L3. The correlation was very strong in both non-sarcopenic HNC patients and healthy adults (r > 0.8), whereas the relationship was fair in sarcopenic patients (r = 0.5712).

**Table 4:** Correlation between actual SMM and predicted SMM at L3 in each group.

	L3 SMM vs. predicted SMM			L3 SMI vs. predicted SMI		
	r	*p-*value	CI	r	*p-*value	CI
Healthy adults	0.9118	< 0.0001	0.8410 to 0.9519	0.8441	< 0.0001	0.7265 to 0.9136
Non-sarcopenia	0.9290	< 0.0001	0.8968 to 0.9514	0.8373	< 0.0001	0.7687 to 0.8869
Sarcopenia	0.7633	< 0.0001	0.6332 to 0.8515	0.5712	< 0.0001	0.3730 to 0.7195

**Fig 5:**
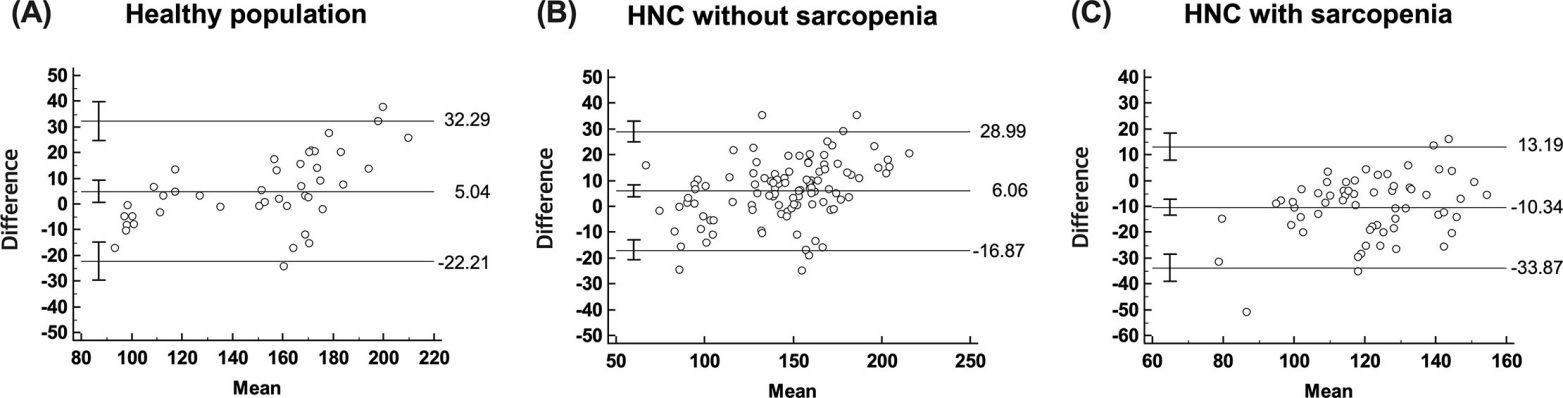
Bland-Altman plot showing the difference against the mean of actual and predicted L3 skeletal muscle masses in each group. Lines indicate the mean and the limits of agreement. HNC, head and neck cancer.

**Fig 6:**
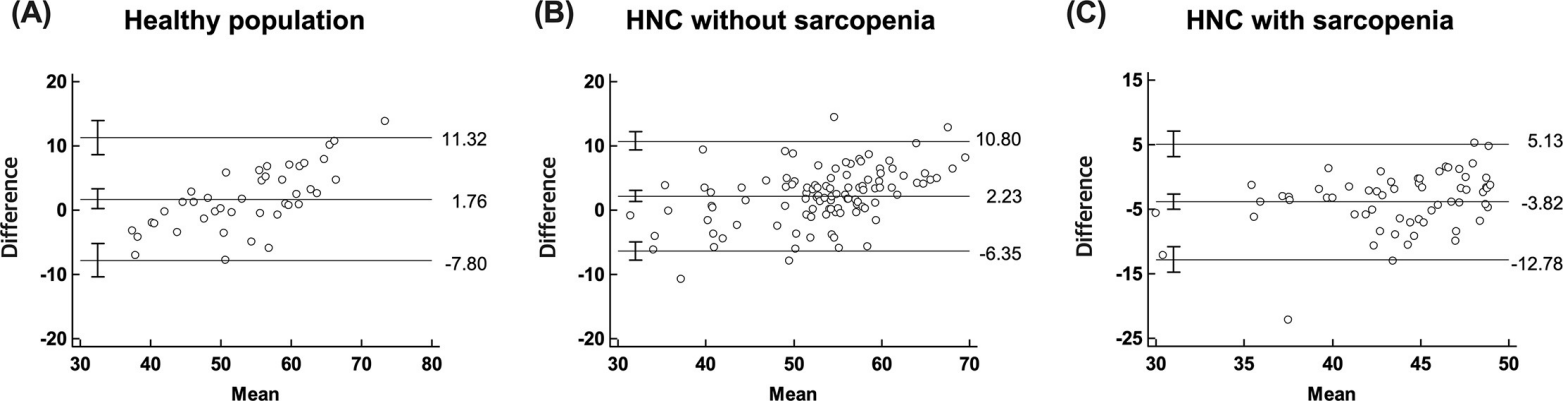
Bland-Altman plot showing the difference against the mean of actual and predicted L3 skeletal muscle indices in each group. Lines indicate the mean and the limits of agreement. HNC, head and neck cancer.

Figs 5 and 6 display the LOAs of differences between 2 measurements for L3 SMMs and L3 SMIs. For L3 SMM, the LOAs ranged from -22.21 to 32.29 with a mean of 5.04 for healthy subjects, -16.87 to 28.99 with a mean of 6.06 for non-sarcopenic patients, and -33.87 to 13.19 with a mean of -10.34 for sarcopenic patients (Fig 5). The agreement was “good” for healthy subjects (2SD = 66.79) and for non-sarcopenic HNC patients (2SD = 63.20). On the other hand, the agreement was “poor” for sarcopenic HNC patients (2SD = 23.45). For L3 SMI, the LOAs ranged from -7.80 to 11.32 with a mean of 1.76 for healthy subjects, -6.35 to 10.80 with a mean of 2.23 for non-sarcopenic patients, and -12.78 to 5.13 with a mean of -3.82 for sarcopenic patients (Fig 6). The agreement was “good” for healthy subjects (3SD = 26.67) and for non-sarcopenic HNC patients (3SD = 23.86). On the other hand, the agreement was “poor” for sarcopenic HNC patients (3SD = 13.50).

### ROC analysis using predicted SMM and SMI at L3 for sarcopenia in HNC patients

**Fig 7:**
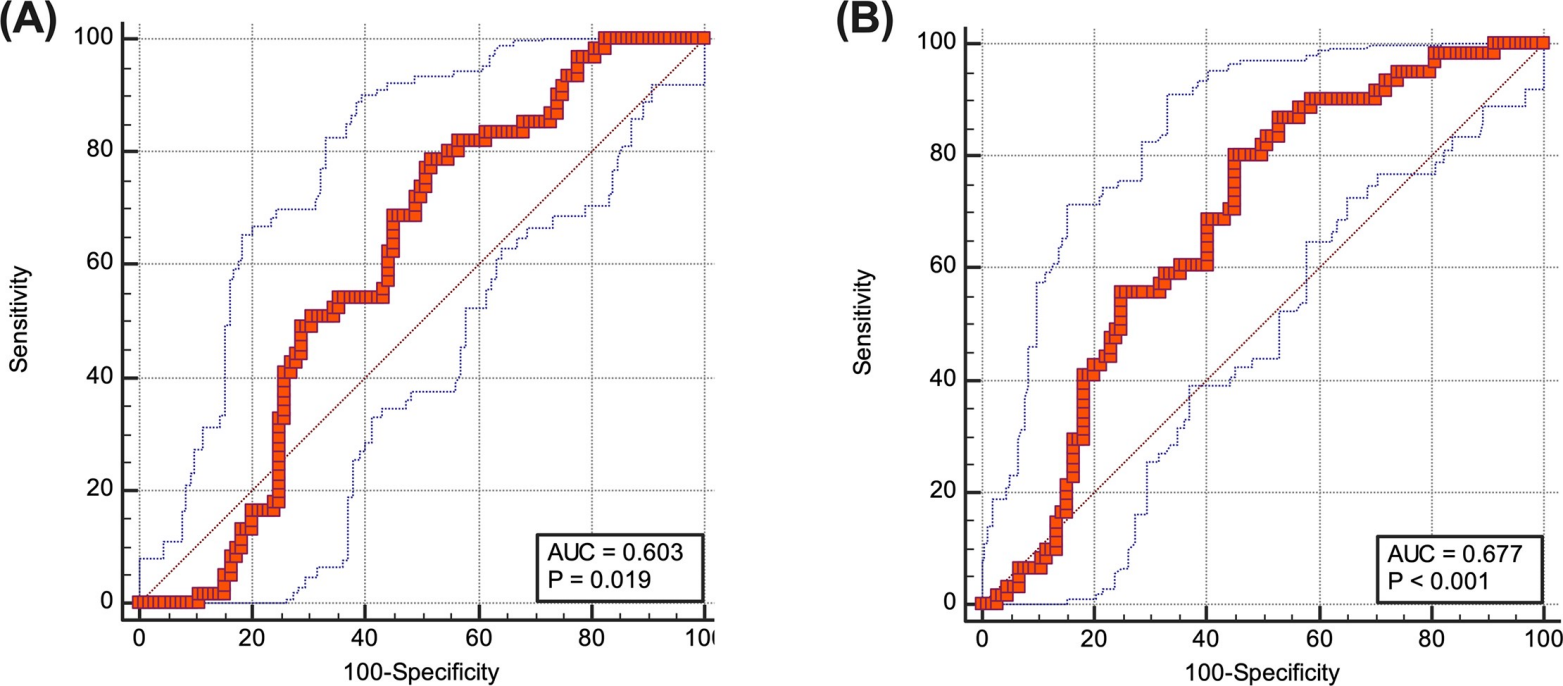
Receiver operating characteristics curve for prediction of skeletal muscle mass at L3 (A) and skeletal muscle index at L3 (B) using paravertebral muscle area at C3 in head and neck cancer patients.

ROC analysis was done to evaluate the diagnostic accuracy of predicted SMM and SMI at L3 for sarcopenia in HNC patients. The AUC of predicted L3 SMM was 0.603 (Fig 7A) and that of predicted L3 SMI was 0.677 (Fig 7B). Although there was a moderate to strong correlation between actual and predicted skeletal mass at L3, the diagnostic accuracy of predicted parameters for sarcopenia was poor (0.6–0.7).

## Discussion

The present study demonstrated a strong relationship between C3 SMM and L3 SMM in both non-sarcopenic patients and healthy controls whereas a weaker relationship in sarcopenic patients with HNC. To our knowledge, this is the first study to show differences in this association in the subgroup analysis of sarcopenic and non-sarcopenic patients.

Nutritional deficiencies and weight loss are common in patients with HNC. These patients experience dysphagia due to tumor location and tumor effects. Many patients with HNC experience poor health and chronic malnutrition [25]. In the present study, 37% of HNC patients were sarcopenic at the time of cancer diagnosis; this rate was approximately 48% in another study [26]. Therefore, HNC patients have a high risk of sarcopenia.

One previous report evaluated the feasibility of using head and neck CT to assess SMM in HNC patients [13] and found a strong relationship between L3 SMM and C3 SMM (r = 0.785) in a cohort of 52 HNC and 51 trauma patients. This result may be attributed in part to a high proportion of non-sarcopenic subjects in their cohort, as their analyses were performed in a single population consisting of both HNC and trauma patients. Mean BMI was normal in both groups and not significantly different between groups, indicating that many patients may have been non-sarcopenic in their study. When re-evaluating our study without subgroup analysis, the relationship between L3 SMM and C3 SMM showed a strong correlation (r = 0.762) in all HNC patients. Another study evaluating 159 HNC patients found a strong correlation between L3 SMI and C3 SMI (r = 0.877, by Spearman correlation); however, a subgroup analysis using only sarcopenic patients was not performed [14].

We developed a prediction model using age, sex, BW and C3 SMM to estimate L3 SMM and SMI. Height was included on initial analysis because it is known to be related to sarcopenia [27]. However, analysis revealed that height was not related to sarcopenia in our population (p = 0.5118), therefore, it was excluded from the equation. A few previous studies developed prediction models to estimate L3 SMM using C3 SMM, and they showed a strong correlation between them in HNC patients; however, subgroup analyses were not performed in them [3,13]. Our prediction model demonstrated that the predicted SMM has a very strong correlation with actual SMM in non-sarcopenic patients, but a moderate correlation in sarcopenic patients. In addition, the agreement by LOAs was “poor” for L3 SMM and L3 SMI in sarcopenic patients. This result indicates that the pattern of skeletal mass loss in sarcopenia may differ between at C3 and at L3. On this assumption, we performed ROC analyses to evaluate whether predicted L3 SMM or its index has comparable diagnostic accuracy for sarcopenia to L3 SMM. As a result, both parameters showed poor diagnostic accuracy (AUC = 0.6–0.7) for sarcopenia determined by L3 SMM, which means that C3 SMM is not an alternative marker to L3 SMM in daily practice. It has been known that FDG PET/CT is a useful imaging tool in staging, planning treatment and monitoring treatment response in advanced HNC. Non-contrast torso CT is available either at the time of diagnosis or during follow-up. Our recommendation is to use L3 SMM on PET/CT instead of using C3 SMM on head and neck CT in evaluating sarcopenia.

We measured only the PVM area at C3 to assess cervical muscle mass. Our finding of a strong relationship between C3 PVM and L3 SMM in our non-sarcopenic cohort was consistent with that of a previous study [13], which found a similarly significant correlation between these variables (r = 0.778). We additionally evaluated the correlation between L3 SMM and C3 PVM+SCM in 42 healthy population and 86 HNC patients without cervical lymph nodes metastasis. All of them were available for the assessment of both SCM on PET/CT. In healthy subjects, the correlation was strong (r = 0.855). The correlation between L3 SMM and C3 PVM+SCM was strong (r = 0.891) in 51 non-sarcopenic patients, whereas the correlation was fair (r = 0.499) in 35 sarcopenic patients. These results were comparable to the results measuring only PVM at C3. Given that assessment of the SCM muscle is often difficult in HNC patients due to local lymph node stations, measurement of only the PVM area may be a suitable alternative in HNC patients without sarcopenia. Our prediction model using C3 PVM demonstrated better correlation coefficients compared with the results using both PVM and SCM muscle which was recently reported in HNC patients [3].

Our study had a couple of limitations. First, it was conducted in a single center and the number of sarcopenic patients was relatively small. Additionally, we used Korean-specific cut-offs to define sarcopenia, which differed from those of Western countries. An international consensus of cancer cachexia defined sarcopenia as an SMI at L3 < 55 cm^2^ for men and < 39 cm^2^ for women [11]. We re-evaluated the relationship between L3 SMM (or SMI) and C3 SMM (or SMI) using the international cut-off for sarcopenia in our study cohort. The results were comparable with those using the cut-off for the Korean population. The relationship between L3 SMM and C3 SMM was weaker in sarcopenic patients (n = 104, *r = 0*.*560*) than in non-sarcopenic patients (n = 61, *r = 0*.*912*) (S1 Fig). A similar relationship was observed between L3 SMI and C3 SMI (*r = 0*.*396 for sarcopenic patients and r = 0*.*768* for non-sarcopenic patients) (S2 Fig). We also evaluated the correlation between actual SMM (or SMI) and predicted SMM (or SMI) using the international cut-off for sarcopenia (S1 Table). The results were similar to those presented in Table 4. However, further studies with a larger cohort including Western patients are necessary to confirm the generalizability of our results. Finally, the definition of sarcopenia in this study was CT-determined sarcopenia. Assessment of muscle strength or physical performance is important for the diagnosis of sarcopenia, we could not evaluate the patients’ muscle strength or physical performance due to the retrospective nature of this study.

In conclusion, although a significant correlation between L3 and C3 SMM was demonstrated in a non-sarcopenic population, no significant association was found in sarcopenic HNC patients. In addition, predicted SMM showed only a moderate correlation with actual L3 SMM in a sarcopenic group, and a poor diagnostic accuracy for sarcopenia. Therefore, C3 SMM may not be a strong predictor for L3 SMM in sarcopenic patients with HNC.

## Supporting information

S1 Fig Association between the skeletal muscle mass at L3 and that at C3 using international cut-off for sarcopenia. (PDF)

S2 Fig Association between the skeletal muscle index at L3 and that at C3 using international cut-off for sarcopenia. (PDF)

S1 Table Correlation between actual SMM and predicted SMM at L3 using International cut-off for sarcopenia. (PDF)

## Abbreviations

AUC: area under the curve
BMI: body mass index
C3: third lumbar vertebra
C3 SMM: skeletal muscle mass at C3
CT: computed tomography
FDG: fluorodeoxyglucose
HNC: head and neck cancer
L3: third lumbar vertebra
L3 SMM: skeletal muscle mass at L3
MRI: magnetic resonance imaging
PET: positron emission tomography
PVM: paravertebral muscle
ROC: receiver operating characteristics
SCM: sternocleidomastoid
SMA: skeletal muscle area
SMI: skeletal muscle index
SMM: skeletal muscle mass
