# Supplementary figures and images for: Skeletal muscle mass at C3 may not be a strong predictor for skeletal muscle mass at L3 in sarcopenic patients with head and neck cancer
Source: PLoS One. 2021 Jul 19;16(7):e0254844. doi: 10.1371/journal.pone.0254844 (PMC8289025; doi:10.1371/journal.pone.0254844)

(A) HNC without sarcopenia

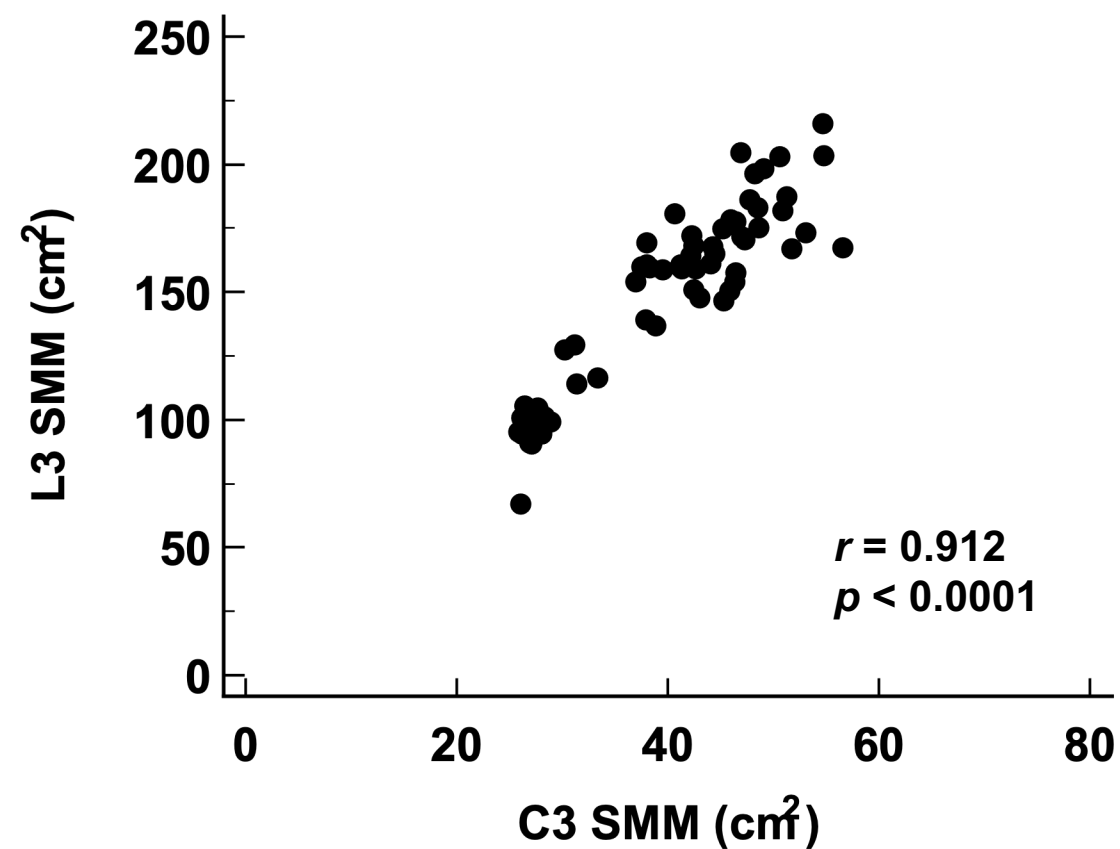

(B) HNC with sarcopenia

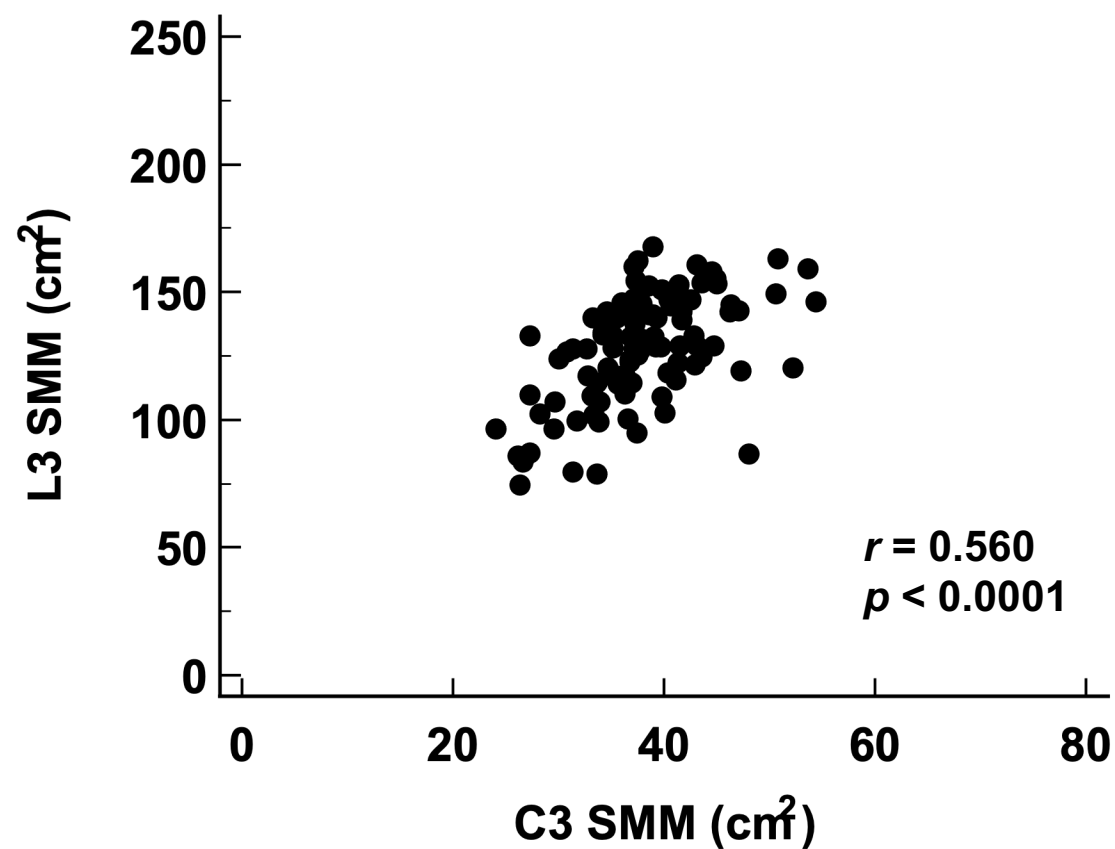

Supplement: S1 Fig — (PDF) [file pone.0254844.s001.pdf]

(A) HNC without sarcopenia

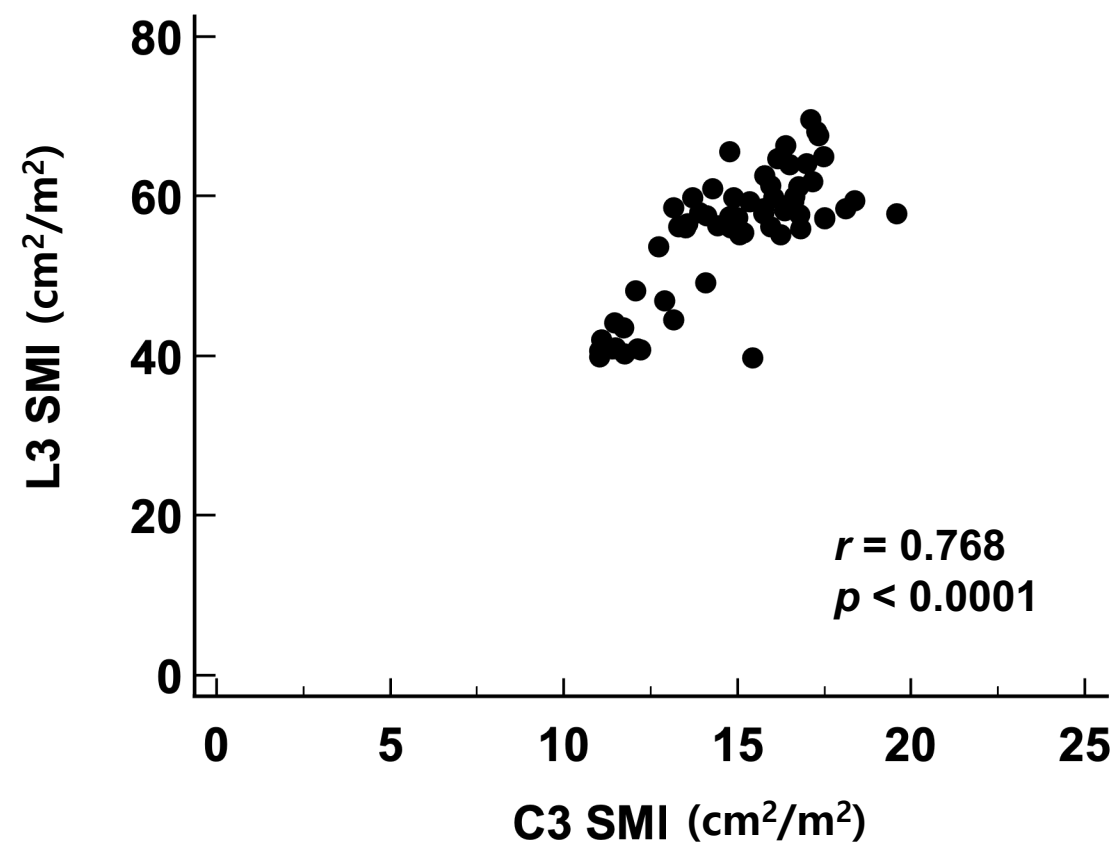

(B) HNC with sarcopenia

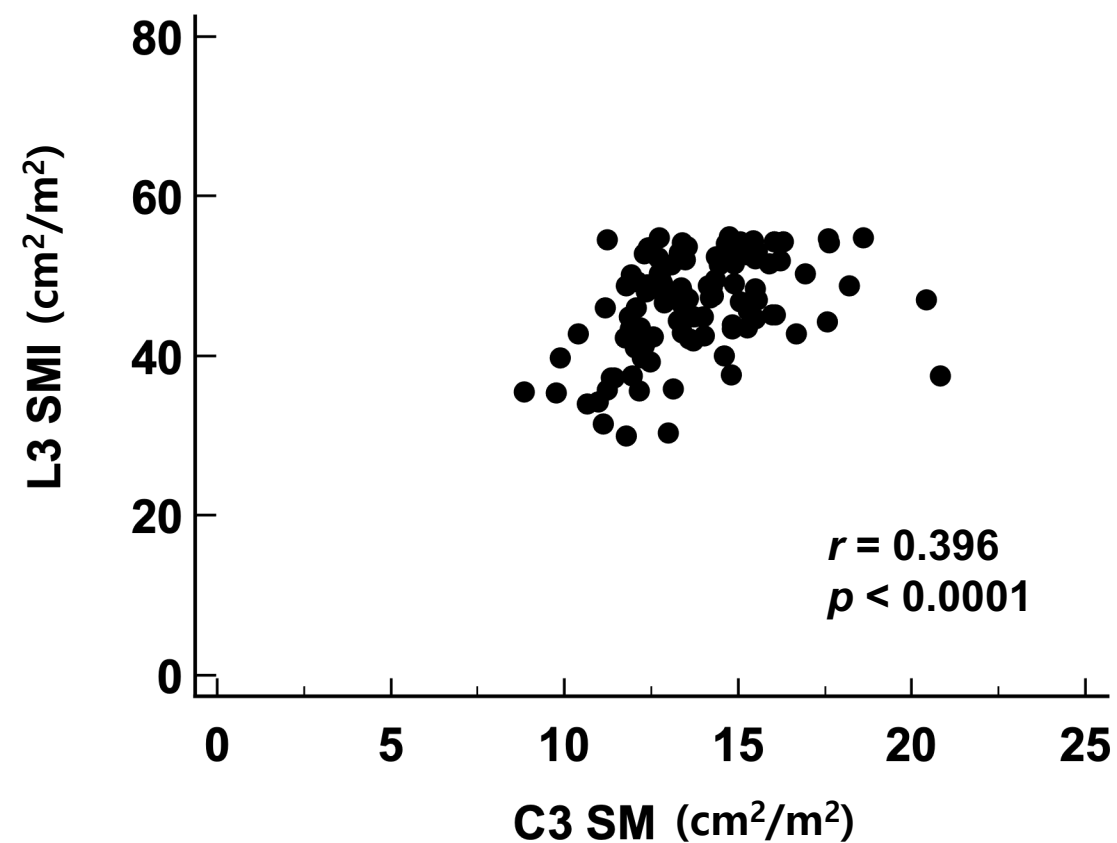

Supplement: S2 Fig — (PDF) [file pone.0254844.s002.pdf]
