# Supplementary material for: Skeletal muscle mass at C3 may not be a strong predictor for skeletal muscle mass at L3 in sarcopenic patients with head and neck cancer
Source: PLoS One. 2021 Jul 19;16(7):e0254844. doi: 10.1371/journal.pone.0254844 (PMC8289025; doi:10.1371/journal.pone.0254844)
Supplement: S1 Table — (PDF) [file pone.0254844.s003.pdf]

Supplementary table 1. Correlation between actual SMM and predicted SMM at L3 in each group (International cutoff)

	L3 SMM vs. predicted SMM			L3 SMI vs. predicted SMI		
	r	*p*-value	CI	r	*p*-value	CI
Non-sarcopenia	0.9512	< 0.0001	0.9197 to 0.9706	0.8664	< 0.0001	0.7861 to 0.9179
Sarcopenia	0.7852	< 0.0001	0.6982 to 0.8493	0.6309	< 0.0001	0.4990 to 0.7343
